# Resistance and Recalcitrance in Dermatophytosis: Mechanistic and Clinical Considerations for Keratinized Tissues

**DOI:** 10.3390/antibiotics15070634

**Published:** 2026-06-24

**Authors:** Alfredo Valdez-Martinez, Roberto Arenas, Andrea Moreno-Salinas, Mariana Perez-Tristan, Maria Jose Gomez-Rico, Ivette Torres-Olguín, Claudia Erika Fuentes-Venado, Fernando Bastida-González, Erick Martínez-Herrera, Rodolfo Pinto-Almazán

**Affiliations:** 1 Sección de Micología, Hospital General “Dr. Manuel Gea González”, Mexico City 14080, Mexico; alfredovaldezmart@gmail.com (A.V.-M.); rarenas98@hotmail.com (R.A.); 2 Clínica Hospital Constitución, Instituto de Seguridad y Servicios Sociales de los Trabajadores del Estado (ISSSTE), Monterrey 64530, Mexico; andrea.moreno2593@gmail.com (A.M.-S.); mariana.perez.tristan@gmail.com (M.P.-T.); maria.jose.gomez.rico@uabc.edu.mx (M.J.G.-R.); ivettetorresolguin@gmail.com (I.T.-O.); 3 Sección de Estudios de Posgrado e Investigación, Escuela Superior de Medicina, Instituto Politécnico Nacional, Plan de San Luis y Díaz Mirón, Mexico City 11340, Mexico; cefvenado@hotmail.com; 4 Servicio de Medicina Física y Rehabilitación, Hospital General de Zona No. 197, Texcoco 56108, Mexico; 5 Laboratorio de Biología Molecular, Laboratorio Estatal de Salud Pública del Estado de México, Toluca de Lerdo 50130, Mexico; mijomeil@hotmail.com; 6 Fundación Vithas, Grupo Hospitalario Vithas, 28043 Madrid, Spain

**Keywords:** dermatophytosis, dermatophytes, antifungal resistance, therapeutic failure, recalcitrant dermatophytosis, terbinafine resistance, *Trichophyton indotineae*, onychomycosis, tinea incognito, dermatophytoma

## Abstract

Dermatophytosis remains one of the most prevalent superficial fungal infections worldwide and is increasingly encountered as a persistent or difficult-to-treat syndrome. A major clinical problem is that apparent treatment failure is often attributed to antifungal resistance, although many cases are instead driven by diagnostic uncertainty, corticosteroid-modified disease, reinfection, inadequate exposure, poor adherence, and limited drug delivery within keratinized tissues. This narrative review was developed to clarify the distinction between true antifungal resistance and clinical recalcitrance, with particular attention to terbinafine-resistant *Trichophyton* species, *Trichophyton indotineae*, tinea incognito, onychomycosis, dermatophytoma, and high-barrier skin and nail infections. We synthesized peer-reviewed literature and guideline-level evidence addressing epidemiology, molecular mechanisms of resistance, clinical phenotypes of recalcitrance, diagnostic escalation, therapeutic decision-making, and antifungal delivery in keratinized tissues. The review contributes a dermatology-centered conceptual framework in which persistent dermatophytosis is interpreted through both microbiological resistance and modifiable recalcitrance drivers. This approach emphasizes confirmation of fungal disease when indicated, phenotypic and anatomic classification, avoidance of inappropriate corticosteroid combinations, optimization of dose, duration, vehicle, and adherence, measures to improve drug access and reduce protected fungal burden in high-barrier disease, and prevention of reinfection from reservoirs. The proposed framework may support more rational antifungal use and reduce unnecessary escalation; however, it is based on narrative synthesis rather than a systematic review or prospective validation. Additional studies are needed to determine how such structured clinical approaches affect clinical outcomes, relapse rates, antifungal exposure, and resistance emergence in real-world dermatology practice.

## 1. Introduction

Superficial dermatophyte infections of the skin and nails remain among the most common mycoses worldwide and represent a substantial everyday burden in dermatology practice, with consequences that extend beyond “minor” disease, including psychosocial impact, recurrent healthcare utilization, and prolonged self-directed therapy [1,2]. In parallel with their ubiquity, dermatophytoses are increasingly encountered as recalcitrant infections, characterized by persistence, rapid relapse, or repeated incomplete responses despite apparently appropriate antifungal therapy. Crucially, recalcitrance is not synonymous with antifungal resistance: it often reflects a convergence of diagnostic uncertainty, iatrogenic lesion modification, reinfection dynamics, and tissue-level barriers that prevent adequate antifungal exposure at the fungal niche [1,3].

This distinction has become more clinically consequential in the last decade, as true resistance has emerged alongside an expanding clinical “failure phenotype”. In multiple regions, outbreaks of extensive and inflammatory tinea have been linked to terbinafine-resistant *Trichophyton* within the *T. mentagrophytes*/*T. interdigitale* species complex and to the global emergence of *Trichophyton indotineae* (formerly genotype VIII), which is frequently associated with elevated terbinafine minimum inhibitory concentrations (MICs) and treatment-refractory courses [4,5,6,7]. Contemporary surveillance in North America has also documented terbinafine-resistant dermatophytes and the presence of *T. indotineae*, underscoring that resistant dermatophytosis is no longer geographically confined and is likely underrecognized because routine speciation and susceptibility testing remain limited [8,9]. However, focusing on resistance alone may misclassify many treatment failures. Recalcitrant presentations may also result from inadequate or interrupted regimens, vehicle mismatch, delayed confirmation of fungal etiology, and ongoing reinoculation from household contacts, shared fomites, or concomitant dermatophyte infections at other body sites [1,2].

Iatrogenic drivers are particularly important in the current era. Tinea incognito—steroid-modified dermatophytosis—can present with atypical morphology, delayed recognition, wider anatomic spread, and repeated cycles of empiric therapy, thereby increasing antifungal exposure while obscuring the true diagnosis [10,11]. Widespread availability and misuse of topical fixed-dose antifungal–corticosteroid combinations have been implicated as accelerators of this cycle in high-burden settings, contributing both to diagnostic delay and to erratic antifungal exposure that can phenocopy resistance and facilitate chronicity [6,8,10].

Beyond behavior and diagnosis, barrier biology and structured infection patterns contribute to recalcitrance, particularly in keratinized tissues. Nail and skin diseases illustrate the problem: even when the organism is not intrinsically resistant, compact fungal masses, including dermatophytomas and other fungal conglomerates, as well as biofilm-like or aggregate phenotypes reported in experimental and clinical contexts, can create diffusion-limited microenvironments in which inadequate drug delivery determines treatment outcome [12]. This limitation is particularly relevant to topical antifungal therapy, where clinical success depends on adequate penetration through keratinized barriers and sustained local exposure at the fungal niche. In nail disease, these barriers may also reduce the apparent effectiveness of systemic therapy if compact fungal biomass or subungual architecture prevents adequate exposure of viable fungal elements [12]. In these contexts, switching antifungal classes alone may fail unless it is paired with measures that improve local drug access and reduce protected fungal burden, such as targeted debridement, drilling or fenestration, keratolysis, and formulation or vehicle choices that optimize sustained exposure at the site of infection [12]. Recent case-based and regional reports of recalcitrant *T. indotineae* infections further reinforce the need to interpret treatment failure through a dual lens: true genetic resistance versus exposure failure, because the corrective actions differ fundamentally [5,13].

These converging trends require a more structured clinical approach to persistent dermatophytosis. Recent calls emphasize that management of superficial mycoses should confirm fungal etiology when feasible, classify syndrome severity and site-specific barriers, verify exposure adequacy and confounders such as corticosteroid use or reinfection, and escalate diagnostics and therapy rationally rather than recycling empiric regimens [1,2]. In India, the formalization of “recalcitrant dermatophytosis” in consensus/position statements reflects the maturation of this concept from anecdote to an actionable clinical problem requiring standardized approaches [14]. A dermatology-focused synthesis is therefore needed to clarify the resistance–recalcitrance distinction, link clinical phenotypes to diagnostic and therapeutic considerations, and frame innovation not only as new molecules but also as delivery science, regimen design, and barrier-directed interventions for keratinized tissues.

The specific problem addressed by this review is the clinical overinterpretation of treatment failure as antifungal resistance without first excluding modifiable causes of recalcitrance. This topic was selected because the global emergence of terbinafine-resistant *Trichophyton* species, particularly *T. indotineae*, has occurred alongside widespread corticosteroid misuse, recurrent empirical antifungal exposure, and persistent diagnostic limitations in routine dermatology practice. This review synthesizes these parallel developments into a mechanistic and clinical framework that may help clinicians decide when to confirm infection, when to suspect resistance, when to escalate diagnostics, and when to correct exposure, adherence, anatomic barriers, or reinfection before switching antifungal classes.

Concept box. Resistance vs. recalcitrance in dermatophytosis.

- Antifungal resistance is a microbiological phenotype in which a dermatophyte exhibits reduced susceptibility to an antifungal agent—typically mediated by stable genetic changes, such as squalene epoxidase (SQLE) gene mutations associated with terbinafine resistance—and is expected to compromise clinical response despite adequate dosing, duration, and adherence.
- Recalcitrance is a clinical phenotype characterized by persistence, repeated partial responses, or rapid relapse of dermatophytosis despite apparently appropriate therapy. It may occur with resistant or fully susceptible isolates and is frequently driven by non-resistance factors, including exposure failure, barrier-limited delivery in keratinized tissues, structured fungal burdens such as dermatophytoma or aggregate-like phenotypes, diagnostic delay or misclassification, steroid-modified tinea incognito, suboptimal regimen execution, and reinfection from contacts or fomites.

Topical versus systemic antifungal therapy: delivery, exposure, and safety considerations.

Topical and systemic antifungal therapies face different delivery and safety constraints. Topical therapy can achieve high local concentrations with minimal systemic toxicity and is appropriate for many limited superficial infections; however, its effectiveness depends on adequate contact time, vehicle selection, adherence, and penetration through keratinized barriers. Hyperkeratosis, occlusion, onycholysis, dermatophytoma, and compact fungal aggregates may prevent sufficient topical drug from reaching viable fungi. Systemic therapy may be required for extensive, inflammatory, follicular, recurrent, or nail disease because it can provide drug exposure beyond the superficial surface, but it is limited by absorption variability, drug–drug interactions, systemic toxicity, contraindications, and the need for adequate duration and adherence. Therefore, apparent treatment failure should be interpreted according to the route of administration: topical failure often reflects inadequate penetration or local exposure, whereas systemic failure may reflect resistance, insufficient duration, poor adherence, pharmacokinetic limitations, or persistent anatomic reservoirs [1,12,14].

As a narrative review, this article synthesizes peer-reviewed and guideline-level literature across five clinically relevant domains: epidemiology and burden; mechanisms of terbinafine and azole resistance; phenotypes associated with recalcitrant dermatophytosis; diagnostic and therapeutic considerations in outpatient dermatology; and antifungal innovation relevant to keratinized tissues, particularly skin and nail disease.

## 2. Burden and Heterogeneity

Global estimates from the Global Burden of Disease (GBD) 2021 analyses consistently rank fungal skin diseases among the most common human infections worldwide, while also demonstrating marked geographic and sociodemographic heterogeneity in incidence, prevalence, and disability burden [15,16]. This variability reflects climate and built-environment determinants that shape exposure and transmission, including heat, humidity, housing density, access to hygiene and healthcare, as well as close-contact settings and occupational or ecological interfaces that increase inoculum pressure, such as schools, sports teams, military or institutional living, and animal contact [15,16]. Importantly, “burden” is not fully captured by point prevalence: dermatophytosis often behaves as a communicable, re-exposure-driven condition within households and teams, where recurrent transmission and interrupted or partial treatment can create cycles of persistence or relapse even in the absence of true resistance [16].

**Figure 1:**
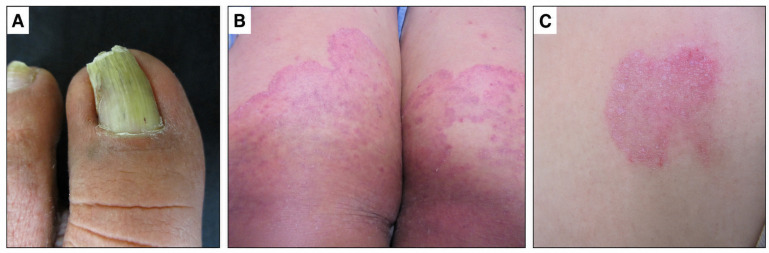
Clinical features of recalcitrant dermatophytosis. (**A**) Onychomycosis presenting with yellow nail discoloration, longitudinal ridging, and subungual hyperkeratosis. (**B**) Corticosteroid-modified dermatophytosis or tinea incognito involving the inguinal-crural region, with extensive erythematous scaly plaques and irregular borders. (**C**) Inflammatory tinea corporis presenting as an annular erythematous plaque with peripheral scaling and partial central clearing. Photographs courtesy of Prof. Dr. Roberto Arenas, used with permission.

The clinical burden is amplified by chronicity and the mismatch between real-life impact and the historic framing of superficial mycoses as trivial. Patient-reported outcome studies document substantial psychosocial and functional impairment in dermatophytosis, with many patients reporting a very large-to-extremely large effect on quality of life and measurable financial strain during prolonged disease courses [17]. Nail disease further concentrates this burden: onychomycosis is chronic, slow to clear, and frequently recurrent, and it can drive pain, footwear limitation, embarrassment, and time-consuming self-care behaviors—domains that consistently register as meaningful quality-of-life losses [18,19]. In high-risk groups such as diabetes, peripheral neuropathy, or vascular disease, nail and foot mycoses become clinically consequential because they can contribute to fissuring, skin breakdown, and downstream bacterial complications; systematic evidence links onychomycosis with diabetic-foot risk profiles and underscores the importance of accurate recognition and effective therapy in these populations [20] (Figure 1).

Heterogeneity also extends to etiology and treatment responsiveness. Species distributions vary by geography and time, and the recent global expansion of terbinafine-resistant *Trichophyton indotineae* illustrates how shifting dermatophyte ecology can reshape clinical phenotypes and outcomes [5,21]. A dermatology-focused approach should therefore consider regional epidemiology and exposure ecology, transmission and re-exposure dynamics, patient-level risk modifiers, and site-specific barrier biology in keratinized tissues, all of which influence whether apparently appropriate therapy achieves adequate drug exposure at the fungal niche [15,16,19,20].

Persistent dermatophytosis is best understood as a multifactorial clinical syndrome in which microbiological resistance may coexist with non-resistance drivers, including prior antifungal exposure, corticosteroid-modified disease, barrier-limited pharmacology in keratinized tissues, reinfection, and pathogen shifts such as the emergence of terbinafine-resistant *Trichophyton*, including *T. indotineae* [5,10,11,14,22,23,24,25]. This distinction matters clinically because treatment failure may otherwise be attributed to resistance alone, leading to premature escalation, unnecessary polypharmacy, or prolonged systemic courses without correction of the dominant driver, such as steroid-masked disease, inadequate exposure or penetration, regimen inconsistency, or persistent reservoirs that enable relapse [10,11,13,14,26,27].

### 2.1. Selection Pressure from Widespread Allylamine and Azole Exposure

In high-burden settings, terbinafine and azoles are often used empirically, repeatedly, and in non-standard regimens, creating conditions that may facilitate selection of less susceptible strains and persistence of infection [13,14,22,23,27]. This pressure is not limited to clinician-directed prescriptions; self-medication, incomplete courses, and inconsistent regimens can yield cycles of partial improvement followed by relapse, which may mimic resistance while reflecting inadequate duration, adherence gaps, misdiagnosis, or reinoculation [13,14,22,27]. Accordingly, escalation without confirmation of fungal etiology or assessment of site-specific pharmacology may expand antifungal exposure without resolving the underlying cause of persistence [14,27].

### 2.2. Topical Corticosteroid Misuse and Tinea Incognito

Topical corticosteroids are often used, either alone or in fixed-dose combinations, because they rapidly reduce pruritus, erythema, burning, and inflammatory scaling. This symptomatic improvement may create the false impression of therapeutic success. However, by suppressing local inflammation and visible border activity, corticosteroids may impair clinical recognition and local host control of dermatophyte infection. As inflammation is blunted, fungal proliferation may continue with less clinical recognition, producing atypical, extensive, or recurrent lesions. This mechanism explains why corticosteroid-containing combinations may temporarily alleviate symptoms while simultaneously delaying diagnosis, increasing fungal burden, promoting tinea incognito, and contributing to repeated or inadequate antifungal exposure [11,23,26,28].

### 2.3. Structured Infections and Barrier Biology: Why Drugs Fail to Reach the Target

Outcomes are strongly shaped by anatomic and microenvironmental constraints that determine whether drug exposure is sufficient at the fungal niche. In skin disease, hyperkeratosis, thickened stratum corneum, occlusion, and follicular involvement can create protected compartments where effective drug concentrations are harder to achieve despite apparently appropriate agent selection [12]. In nail disease, several structural constraints converge, including slow nail growth, limited vascular contribution to the nail plate, onycholysis with a sheltered subungual space, and compact fungal masses such as dermatophytoma or aggregate-like phenotypes that may behave as diffusion-limited systems in selected contexts [12,29]. These features can reduce penetration and slow fungicidal kinetics, providing a mechanistic rationale for combined strategies, such as mechanical or physical debulking plus topical or systemic therapy, to reduce fungal burden and improve drug access in recalcitrant nail infections [12,24]. In this model, recalcitrance may be driven less by intrinsic resistance than by pharmacologic exclusion, meaning insufficient concentration or duration at protected microenvironments where viable fungal elements persist [12,24].

### 2.4. Pathogen Shifts and the Emergence of Terbinafine-Resistant Trichophyton, Including T. indotineae

The emergence and spread of terbinafine-resistant *Trichophyton*, particularly strains now recognized as *T. indotineae*, has been documented across multiple regions and increasingly detected beyond the settings where they were first recognized [5,13,25]. This shift can disrupt standard treatment expectations: terbinafine may fail where resistance prevalence is substantial, lowering the threshold for diagnostic confirmation, species-level identification, and consideration of alternative systemic agents guided by local epidemiology and, when available, susceptibility testing [5,13,25]. Importantly, pathogen emergence intersects with other drivers of persistence, including antifungal overexposure, corticosteroid-modified disease, and barrier-limited drug access in keratinized tissues [14,22,23,24,25,26,27]. A clinically useful approach should therefore separate true resistance, pseudo-failure due to delivery barriers or regimen mismatch, and relapse due to persistent reservoirs or reinfection, because each requires a different corrective action [14,25,27].

## 3. Resistance Mechanisms in Dermatophytes: From Gene to Bedside

Dermatophyte treatment failure is clinically useful when framed as a continuum rather than as a binary susceptible/resistant label [30,31]. At one end are stable genetic mechanisms that raise minimum inhibitory concentrations and may predict drug failure despite adequate exposure, with terbinafine resistance linked to squalene epoxidase changes and resistant lineages as the classic example [5,32,33,34]. Between genetic resistance and purely clinical failure are adaptive tolerance states, in which stress-response remodeling and transient survival programs may allow persistence under drug pressure without a single defining mutation. In *Trichophyton rubrum*, tolerance to terbinafine has been linked to signaling and ion-homeostasis circuitry, such as Ptk2–Pma1, illustrating that non-minimum inhibitory concentration mechanisms can influence persistence [35]. At the exposure-related end of the spectrum, apparent failure may occur when effective drug concentrations at the fungal niche are inadequate because of keratinized-tissue barriers, aggregate-like growth patterns, or regimen mismatch involving vehicle, duration, or adherence [36,37].

Although terbinafine resistance remains the best-characterized paradigm in dermatophytes, azole resistance has also been reported, including increased itraconazole resistance associated with Erg11A point mutations in *Trichophyton quinckeanum* [38].

This continuum is clinically relevant because different mechanisms imply different corrective actions. Genetic resistance should prompt species or epidemiologic-context clarification and, when feasible, antifungal susceptibility testing and rational class switching. Tolerance or pseudo-resistance calls for optimization of dose, duration, adherence, and avoidance of repeated short-cycle therapy. Exposure-related failure requires barrier-aware delivery and measures that reduce protected fungal burden and improve drug access, rather than simply stacking systemic agents [30,31,39,40,41,42,43]. A structured clinical approach can help identify which failure patterns should prompt mycologic confirmation, species identification, susceptibility testing, referral, and/or treatment modification [30,31,40,42,44]. A pragmatic diagnostic–therapeutic loop is: confirm fungal disease, identify the likely pathogen and resistance context, verify adequate exposure, and escalate rationally [30,32,36,45] Table 1.

### 3.1. Allylamines (Terbinafine): SQLE as the Dominant Axis

Terbinafine inhibits squalene epoxidase (SQLE) in the ergosterol pathway. Across *Trichophyton*, SQLE-linked resistance remains the dominant and most clinically actionable molecular axis, correlating with elevated terbinafine MICs and treatment failure under otherwise adequate dosing and adherence [31,32,33,45]. The clinical relevance of this mechanism has increased as terbinafine-resistant lineages, particularly *Trichophyton indotineae*, have expanded geographically and begun to appear outside initial high-burden epicenters, raising the pre-test probability that true resistance underlies a subset of refractory cases [3,4,5,6,7,8,9,13,21,25,30,40,41,45].

When does terbinafine failure become a resistance signal rather than nonspecific treatment failure? Because many failures reflect confounders such as diagnostic error, inconsistent exposure, incomplete courses, steroid modification, or ongoing transmission, a structured clinical approach benefits from defining higher-suspicion scenarios in which resistance work-up is justified [1,3,10,11,14,30,39,40]. Suspicion is higher when:

- Inflammatory and/or extensive disease persists or worsens despite appropriately dosed terbinafine with credible adherence [3,4,5,6,7,8,9,13,30,32,33,40,41,45].
- Mycologic confirmation is documented (KOH/culture/PCR) and remains positive during or after adequate therapy [3,14,30,39,40,41].
- The epidemiologic context suggests elevated risk, such as local reports of resistant dermatophytes, contact/travel patterns compatible with *T. indotineae*, or clustering of refractory cases [3,4,5,6,7,8,9,13,21,25,30,40,41,45].

Clinical translation.

Persistent inflammatory tinea after adequately dosed terbinafine should prompt consideration of:

- Confirmation of fungal etiology before repeating or stacking therapies (KOH ± culture/PCR) [1,3,14,30,39,40].
- Identification of the organism or species complex when feasible, because *T. indotineae* materially changes treatment expectations and escalation thresholds [3,4,5,6,7,8,9,13,21,25,30,39,40,41,45].
- Optimization of exposure followed by rational escalation: avoid serial short courses; consider switching to a systemic azole guided by clinical context, drug interactions, and local epidemiology, often with topical adjuncts and barrier-aware delivery; and pursue susceptibility testing where available to support agent selection and dosing [30,31,33,39,40,44].

In practical terms, treatment failure becomes meaningful as a resistance signal only after fungal certainty and exposure adequacy have been assessed. Otherwise, escalation risks widening antifungal pressure without correcting the reason the infection persists [1,3,14,30,39,40].

### 3.2. Azoles: ERG11/CYP51 Alterations, Efflux/Tolerance, and Cross-Resistance Concerns

Azoles inhibit lanosterol 14α-demethylase, encoded by ERG11/CYP51, in the ergosterol pathway. In dermatophytes, azole resistance is less standardized than in *Candida*, with fewer harmonized clinical breakpoints and more variable routine testing. Nevertheless, mechanistic and clinical evidence supports roles for target-pathway alterations, increased target gene dosage, and efflux or tolerance programs that reduce effective intracellular drug exposure and enable persistence [3,14,30,31,33,34,35,38,39,40,44]. Notably, CYP51B amplification has been described as a resistance mechanism associated with reduced itraconazole and voriconazole activity in *T. indotineae*, providing a concrete genetic route to azole failure beyond nonspecific tolerance [34]. Additional observations of ERG11/Erg11A-associated changes with increased azole resistance in dermatophyte lineages further support the plausibility of target-driven azole failure under sufficient exposure [38].

How azole “failure” presents in practice.

Azole non-response may reflect:

- Disease extent or morphology that makes topical-only therapy inadequate, such as wide surface area, hyperkeratosis, or follicular involvement [3,14,39,40].
- Regimen mismatch, including underdosing, short cycling, interrupted courses, or poor adherence, which can produce persistence that mimics intrinsic resistance [1,3,14,30,39,40].
- Exposure erosion from systemic pharmacology issues, including drug–drug interactions and variable absorption, which is clinically relevant even if not a fungal mechanism [3,14,39,40].

Cross-resistance, efflux, and the “serial failure” trap.

If tolerance, efflux programs, and/or target alterations contribute to persistence, switching among azoles without correcting exposure or confirming the pathogen can yield serial failures [31,33,34,35,38]. A structured approach therefore emphasizes confirming the organism, avoiding repeated short empiric azole cycles, and matching systemic therapy to severity and delivery constraints—often pairing systemic therapy with topical adjuncts and barrier-aware strategies [1,3,14,30,39,40]. Finally, because azole antifungal susceptibility testing can be method-sensitive in dermatophytes, interpretation of susceptible versus resistant results requires awareness of methodological limitations and the value of standardized approaches, including EUCAST-based frameworks where available [31,33,39,40,44].

### 3.3. Tolerance, Aggregate-like Phenotypes, and Exposure-Related Failure

In dermatology practice, some cases of apparent antifungal failure may reflect exposure-related recalcitrance rather than genetic resistance, particularly when viable fungal elements persist in keratinized compartments where drug access is limited. This is especially apparent in onychomycosis, where slow nail growth, nail plate thickness, subungual hyperkeratosis, and onycholysis create protected microenvironments with reduced drug access, so in vitro susceptibility may coexist with in vivo persistence [12,42,46,47]. Compact fungal masses, such as dermatophytoma, and biofilm-like or aggregate phenotypes reported in experimental and clinical contexts may add a second layer of protection by acting as diffusion barriers or persistence-supporting microhabitats under drug pressure [12,36,37]. Evidence from transungual penetration studies and delivery-focused reviews reinforces that delivery, vehicle, and sustained exposure can influence outcomes in keratinized tissues, particularly when the fungal burden is compartmentalized behind physical barriers [46,47].

Importantly, exposure failure is not confined to nail disease. In cutaneous dermatophytosis, recalcitrance may also emerge when fungi persist within hyperkeratotic, occluded, intertriginous, or hair-bearing sites, where the local microenvironment, follicular extension, moisture, friction, and altered morphology can reduce effective drug exposure or delay recognition of the true syndrome [10,11,14]. In these settings, extensive tinea corporis/cruris, steroid-modified presentations, and recurrent disease linked to household or community reservoirs may phenocopy resistance even when intrinsic reduced susceptibility is absent, because persistence is maintained by diagnostic distortion, inadequate regimen execution, reinoculation, and incomplete control of reservoirs [1,10,11,14,23,26,27,28].

### 3.4. Clinical Translation: Restore Exposure and Reduce Protected Fungal Burden

When barrier biology and aggregate-like growth patterns dominate, interventions that lower fungal biomass and improve drug access may help convert a recalcitrant course into a treatable one. Practical approaches include: (i) targeted mechanical or physical measures, such as atraumatic nail removal, debridement or debulking, and selected device-based approaches used as adjuncts, paired with antifungal therapy rather than used alone [24,42,48]; (ii) penetration-aware topical strategies as adjuncts, even when systemic agents are used, emphasizing appropriate vehicle, consistent long-term application, and, when appropriate, delivery-enhancing formulations [46,47]; and (iii) direct management of anatomic barriers, including onycholysis, hyperkeratosis, and dermatophytoma-like compact masses that may function as diffusion barriers [12,42,49]. In recalcitrant nail disease, delayed response despite an appropriate systemic agent should prompt reassessment of whether effective antifungal concentrations are reaching the fungal niche. If drug access is likely inadequate, class-switch escalation alone may fail unless penetration optimization and reduction in protected fungal burden are added to restore effective exposure at the protected niche [12,24,42,46,47,48,49].

## 4. A Recalcitrance-Centered Diagnostic Approach: Confirm–Classify–Culture/Sequence When Needed

**Figure 2:**
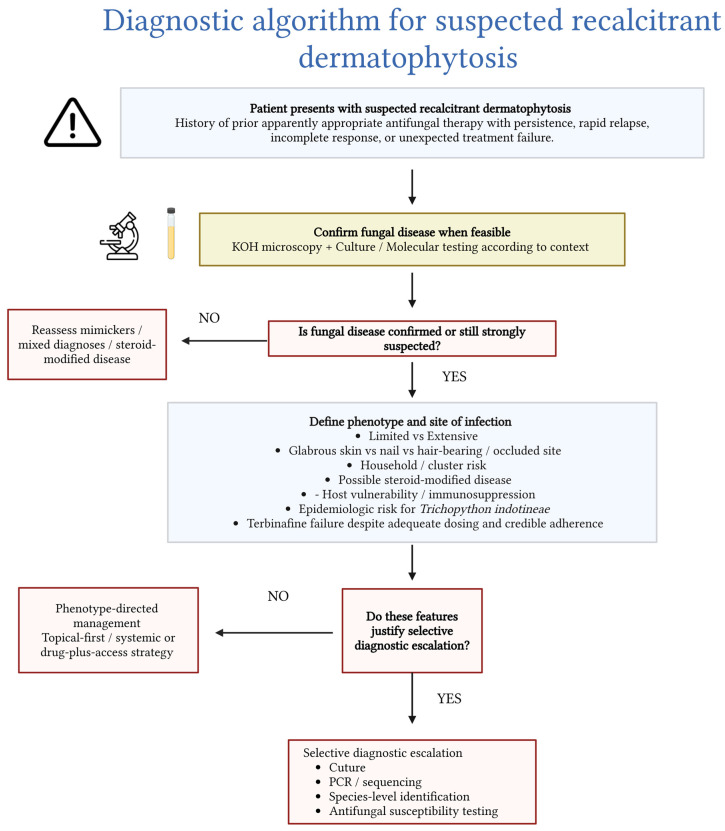
Diagnostic algorithm for suspected recalcitrant dermatophytosis.

In the current resistance era, diagnostic evaluation in dermatology should not be framed as indiscriminate testing, but as a structured response to recalcitrance. The central aim is to distinguish true antifungal resistance from other common causes of persistence, including misdiagnosis, steroid-modified dermatophytosis, reinfection, inadequate drug exposure, and unrecognized epidemiologic risk [1,11,39]. A practical approach is therefore: Confirm–Classify–Culture/Sequence when needed. This model supports rational antifungal use while reducing both empirical overtreatment and delayed recognition of resistant or emerging dermatophyte lineages [1,39,40] Figure 2.

The first step is to confirm fungal disease, ideally before systemic therapy is prescribed. Whenever feasible, suspected dermatophytosis should be verified mycologically by potassium hydroxide (KOH) microscopy or an equivalent rapid method, with culture and/or molecular confirmation added according to disease severity, recurrence, treatment history, and epidemiologic context [1,39]. KOH microscopy is a rapid, low-cost diagnostic technique in which skin scales, nail debris, or hair fragments are treated with potassium hydroxide to digest keratin and facilitate direct microscopic visualization of fungal elements, such as septate hyphae or arthroconidia. This step becomes particularly important in recalcitrant disease, where repeated empirical therapy may perpetuate diagnostic error and unnecessary selective drug pressure [1,11]. Although molecular diagnostics are increasingly relevant, conventional microscopy and culture remain widely used in routine dermatology practice and continue to provide meaningful clinical value, especially when interpreted within a structured diagnostic framework [1,39,50].

Compared with bacterial infections, dermatophyte infections are less consistently investigated with species-level identification and susceptibility testing in routine clinical practice. This gap partly reflects differences in clinical setting, diagnostic urgency, culture turnaround time, laboratory availability, and standardization of susceptibility testing. Dermatophytosis is commonly managed empirically in outpatient dermatology, whereas bacterial infections more often require rapid organism identification and susceptibility-guided treatment decisions. In dermatophyte disease, fungal culture is slower, morphology-based identification requires expertise, molecular identification may not be widely available, and susceptibility testing remains less accessible in routine practice. As a result, resistant dermatophytes may be underdetected, and clinical failure is often interpreted without microbiological confirmation [1,39,40,50].

The second step is to classify the clinical syndrome, not merely to name the organism. Once fungal disease is confirmed or strongly suspected, the clinician should define the phenotype in a way that helps explain why the case may become recalcitrant. Useful categories include localized versus extensive disease, glabrous skin versus hair-bearing or occluded/intertriginous sites, nail involvement, possible tinea incognito, household or community clustering, and host vulnerability such as immunosuppression [1,11,39,40]. This classification is clinically relevant because recalcitrance is often phenotype-driven before it is genotype-driven. For example, steroid-modified tinea may persist because of delayed recognition and inflammatory masking, whereas nail disease may remain refractory because barrier architecture limits drug access even when true microbiological resistance is absent [1,11,12].

The third step is to escalate diagnostics selectively when the phenotype justifies it. Culture, PCR, sequencing, species-level identification, and—where available—antifungal susceptibility testing should be reserved for defined recalcitrant scenarios rather than applied indiscriminately [1,39,40]. Practical triggers include terbinafine failure despite adequate dosing and credible adherence, recurrent or extensive dermatophytosis, exposure contexts compatible with *Trichophyton indotineae*, household or institutional clusters, immunosuppression, or concern for deeper or unusually invasive dermatophyte infection [5,25,39,40,41]. In these settings, diagnostic escalation helps distinguish persistent exposure failure or reinfection from reduced susceptibility and emerging resistant lineages [5,25,39,40].

A practical principle follows from this approach: before changing the drug, clarify the driver of recalcitrance. In many patients, the highest-yield intervention is not an immediate class switch, but confirmation of fungal disease, reclassification of the syndrome, and targeted escalation of laboratory work-up only when the result is likely to change management [1,39,40].

### When to Suspect an Emerging or Resistant Dermatophyte: Trichophyton Indotineae as a Clinical Sentinel

Among emerging dermatophytes, *Trichophyton indotineae* is one of the clearest examples of why a recalcitrance-centered diagnostic approach is clinically relevant. It is associated with extensive, inflammatory, frequently relapsing dermatophytosis and with a substantial burden of terbinafine resistance, with international spread now documented across multiple regions [5,39]. As such, it should be viewed not simply as a geographically restricted organism, but as a clinically important marker of resistant or difficult-to-treat dermatophytosis [5].

In practice, *T. indotineae* should be suspected when disease is extensive, inflammatory, chronic, recurrent, or unexpectedly refractory to terbinafine, particularly in tinea corporis/cruris patterns, in patients with relevant travel or contact history, or in household and community clusters [5,39]. Not every refractory case is caused by *T. indotineae*; rather, this organism should be considered when persistence is disproportionate to the apparent severity of disease or to treatment that would otherwise be expected to succeed [5,39].

Recognition may be delayed by corticosteroid misuse, atypical morphology, and overreliance on empirical treatment [11,39]. In this context, early mycologic confirmation, careful syndrome classification, and timely escalation to species-level identification can reduce diagnostic delay and limit repeated ineffective terbinafine exposure [1,11,39]. The literature on tinea incognito further underscores how steroid-modified presentations can obscure the fungal diagnosis until disease becomes more extensive or chronic [11].

Accordingly, *T. indotineae* is best understood not only as a resistant species, but as part of a broader difficult-to-treat phenotype shaped by the interaction of organism, host, anatomy, and prior treatment practices [1,5,39]. Recognizing that phenotype early allows clinicians to move from repeated empiricism toward targeted diagnostic clarification: confirm the infection, define the context, and escalate laboratory resolution only when species identity or resistance status is likely to alter management [1,39].

## 5. Therapeutic Considerations: Choose Wisely, Treat Adequately, Prevent Relapse

### 5.1. Why Therapeutic Strategy Matters in Dermatophytosis

A structured therapeutic approach is increasingly relevant in dermatology because apparent antifungal “failure” in routine practice may reflect not only resistance, but also recalcitrance driven by misdiagnosis, empirical or inappropriate treatment exposure, steroid-modified presentations, inadequate drug selection, insufficient duration, poor penetration at keratinized sites, and failure to address persistent fungal reservoirs or reinoculation pathways [1,10,11,14,39].

In this setting, treatment decisions should align therapy with syndrome biology, anatomic site, resistance context, and exposure barriers, while reducing avoidable selective pressure and preserving the usefulness of existing antifungal agents [1,3,14,39,40]. This is especially important in outpatient dermatology, where empiric therapy remains common, prior unsupervised exposure is frequent, and access to standardized antifungal susceptibility testing or resistance surveillance remains uneven across settings [1,3,4,39,40].

A recalcitrance-centered therapeutic approach shifts the clinician away from reflexive “drug switching” and toward a more useful question: why is this infection persisting? In many cases, persistence reflects the interaction of host factors, disease extent, anatomic site, fungal burden, nail or keratin architecture, prior corticosteroid exposure, and ongoing reinoculation or undertreated concomitant disease [10,11,12,14,41]. Accordingly, the goal is not only to choose the “right antifungal,” but also to choose the right treatment strategy for the right anatomic compartment, for the right duration, with appropriate adjuncts to reduce fungal burden, improve drug access, and prevent relapse [1,12,14,42,43].

### 5.2. A Structured Clinical Approach

A practical outpatient approach can be organized around six linked actions: confirm when indicated, classify the syndrome, match therapy to site and recalcitrance risk, combine intelligently in high-barrier disease, reduce protected fungal burden, and prevent recurrence through reservoir management [1,14,39,40,42].

First, confirm with KOH, culture, and/or molecular methods when clinically indicated, particularly before systemic escalation, after treatment failure, in atypical or steroid-modified presentations, or when resistant dermatophytes such as *Trichophyton indotineae* are a concern [1,3,10,11,39]. Diagnostic confirmation reduces repeated empirical exposure and helps distinguish dermatophytosis from inflammatory mimickers or corticosteroid-modified presentations such as tinea incognito [1,10,11].

Second, classify severity and site carefully, because therapeutic intensity should follow syndrome biology rather than prescribing habit. Limited superficial disease on glabrous skin may often be managed with topical-first therapy, whereas extensive, inflammatory, recurrent, follicular, scalp-associated, or otherwise recalcitrant disease more often requires systemic treatment or a structured escalation strategy [14,19,39,40,41]. Nail disease deserves separate consideration because onychomycosis, especially when associated with dermatophytoma, marked onycholysis, or subungual hyperkeratosis, behaves as a high-barrier infection in which recalcitrance often reflects poor penetration and protected fungal burden rather than simple drug mismatch [12,42,43,46,48].

Third, choose therapy matched to site and resistance risk. For limited superficial disease, topical therapy remains appropriate when adherence is likely and the infection is uncomplicated [19,42,43]. By contrast, extensive, inflammatory, follicular, or recurrent disease usually warrants systemic therapy, and in the setting of terbinafine failure—particularly when adherence and diagnosis have been reasonably assessed—management should become resistance-aware, with reconsideration of organism identity, prior drug exposure, epidemiologic context, and whether a switch to an alternative systemic class is biologically justified [3,4,8,9,39]. Recent literature on *T. indotineae* and terbinafine-resistant dermatophytosis reinforces that repeated ineffective terbinafine exposure should be avoided once a genuinely recalcitrant or resistance-suspected phenotype has emerged [5,6,13,40,41].

Fourth, combine intelligently in high-barrier infections. In onychomycosis and other structured infections, combination approaches may be more rational than monotherapy because they address both viable fungi and the physical barriers that protect them. Current reviews and guidance support combining local and systemic strategies in selected nail infections, particularly when fungal burden is compartmentalized, the nail plate is thickened, or dermatophytoma is present [12,24,42,43,46]. This does not mean that all combination therapy is automatically superior, but rather that recalcitrant nail disease often requires a strategy that improves local exposure while maintaining systemic activity [12,42,43,46,48].

Fifth, reduce protected fungal burden. In dermatophytoma, severe onycholysis, and hyperkeratotic onychomycosis, treatment may fail if fungal biomass and drug access are not addressed. Debridement, reduction in infected keratin, and selected adjunctive physical approaches can lower protected fungal burden and improve penetration of antifungal therapy [12,24,42,48,49]. Debridement is particularly valuable in dermatophytoma-associated disease, where dense compact fungal masses may behave as diffusion barriers and sustain recalcitrance unless mechanically or physically disrupted [12,43,48,49].

Finally, prevent relapse by addressing reservoirs and reinoculation. Recalcitrant dermatophytosis often persists not because treatment was intrinsically inadequate, but because prior exposure patterns, self-medication, steroid-combination misuse, persistent concomitant infection, or ongoing environmental and behavioral drivers are not addressed [14,22,23,26,28]. A structured clinical approach should therefore include practical relapse prevention: evaluate for concomitant tinea pedis in patients with nail disease, address contaminated footwear or shared personal items when relevant, identify misuse of topical corticosteroid combinations, and reduce persistent reservoirs where feasible [14,22,23,27,42]. In this sense, treatment planning extends beyond prescription choice to include interruption of the clinical and behavioral cycle that allows recurrence to masquerade as resistance [1,14,26,39,40].

For recalcitrant skin disease, treatment planning must also account for determinants that differ from those of nails. In glabrous-skin or intertriginous dermatophytosis, persistence may be driven by large body surface area, partial adherence to prolonged topical regimens, prior corticosteroid exposure, concomitant untreated sites, and repeated reinoculation from close contacts, shared fomites, or community transmission networks [1,10,11,14,22,23,26,27]. Accordingly, management of extensive or recurrent cutaneous disease should not rely on class switching alone, but on re-confirming the diagnosis when needed, identifying steroid-modified or follicular phenotypes, simplifying treatment execution, and addressing reservoirs that sustain relapse [1,14,39,40].

Taken together, therapeutic management in dermatophytosis can be summarized in three linked principles: choose wisely, treat adequately, and prevent relapse. For recalcitrant dermatophytosis, this means not only selecting an antifungal, but also confirming the diagnosis when needed, matching treatment intensity to the clinical phenotype, incorporating combination or barrier-directed approaches for structured disease, and addressing the behavioral and anatomic drivers that sustain recurrence [1,12,14,39,42].

## 6. Antifungal Advances for Keratinized Tissues: Clinically Relevant Considerations in Dermatology

Innovation that changes everyday dermatology practice does not always arrive as a completely new antifungal class. In keratinized tissues, clinically relevant advances may also involve improved delivery, formulations that enhance the performance of known active agents in difficult anatomic sites, and oral options with pharmacokinetic or safety profiles better suited to chronic outpatient care. For the stratum corneum and nail unit, the central problem is not merely choosing an active molecule, but achieving sustained exposure at the fungal niche while minimizing systemic toxicity and supporting adherence over long treatment horizons [14,19,42,46,47]. In this context, clinically meaningful progress often reflects delivery optimization and regimen design rather than novelty of target alone.

### 6.1. Topical Advances and Nail Delivery

Why delivery matters.

The nail unit is a diffusion-limited target: drug must traverse the nail plate and/or reach the subungual space, contend with onycholysis and subungual hyperkeratosis, and maintain exposure long enough to overcome fungal persistence and slow nail growth [19,42,46,47]. Topical advances therefore center on low surface tension and wetting, partitioning into keratin, permeation through or around the nail plate, and patient-acceptable regimens that sustain daily use for months.

Clinical proof-of-concept from pivotal trial programs.

Large pivotal trial programs illustrate that formulation matters and that clinically meaningful endpoints can be reached in distal lateral subungual onychomycosis, even without routine debridement in trial protocols, although real-world practice often benefits from adjunctive nail care in high-burden disease [51].

Efinaconazole 10% solution provides a delivery-forward platform, based on a solution vehicle with favorable nail spreading and wetting, with demonstrated efficacy in distal lateral subungual onychomycosis in pivotal trials. This supports the concept that optimized topical exposure can translate into measurable cure endpoints in onychomycosis [51].

Tavaborole 5% solution, a leucyl-transfer ribonucleic acid synthetase inhibitor, demonstrated efficacy in phase III studies, highlighting that even when the mechanism is newer, clinical success still depends on delivery and long-term adherence in the nail environment [51,52].

Topical terbinafine 10% solution, also known as MOB-015, represents a formulation-led strategy to harness a fungicidal class widely used systemically, aiming to improve nail delivery while minimizing systemic exposure and drug–drug interactions. This may be particularly relevant in older adults and patients with polypharmacy [53].

Clinical relevance of modern topicals.

These agents are not simply alternatives to oral therapy; they may offer advantages in selected patients and anatomic contexts. First, they can reduce systemic toxicity and drug–drug interactions compared with oral regimens, which is valuable when comorbidity is common [14,19,42,46]. Second, once-daily solutions with better cosmetic acceptability and easier application may reduce non-adherence, one of the common causes of apparent treatment failure [14,19,42,54]. Third, topicals can serve as rational adjuncts to systemic therapy or physical approaches in high-barrier disease, such as thick nails, onycholysis, and hyperkeratosis, where pharmacologic reach is a limiting step [12,24,42,43,46,48,54]. For nail disease, topical advances are therefore best judged by whether they improve effective exposure, including penetration and persistence, and whether they remain usable for 36 to 52 weeks or longer in real patients—not by novelty of target alone [14,19,42,46,47,54].

### 6.2. Reducing Protected Fungal Burden Plus Topical Strategy in Dermatophytoma and Recalcitrant Nails

Dermatophytoma illustrates how compact fungal burden and impaired exposure may contribute to recalcitrance independently of, or alongside, intrinsic resistance.

Why physical approaches matter mechanistically.

Interventions such as debridement, drilling or fenestration, chemical softening, and partial avulsion in selected cases can reduce inoculum, remove dense compact material that behaves as a diffusion barrier, decrease nail thickness and keratin burden that limit topical transit, and improve contact between antifungal agents and fungal elements in the subungual space [12,24,42,43,46,48].

Evidence signals and how to interpret them.

Case reports and series describing success with efinaconazole in dermatophytoma contexts are better interpreted as mechanistic and clinical signals than as proof of superiority. When outcomes improve after debridement or targeted nail care is combined with an optimized topical, this supports the concept of exposure-related recalcitrance driven by structure rather than genetics [12,24,36,42,43,46,48]. The clinically actionable message is that dermatophytoma management is not simply “one more drug,” but a drug-plus-access strategy, ideally with objective follow-up using clinical and, when feasible, mycologic endpoints [1,12,24,40,42,43,48].

Direct relevance to compact fungal conglomerates in keratinized tissues.

Compact fungal conglomerates represent a translational bottleneck: when fungi persist within dense keratinized structures, the key clinical question becomes how to restore antifungal exposure. Dermatophytoma is the nail prototype of that problem, and the relevant clinical advances include not only new topicals but also reproducible approaches to reducing protected fungal burden that can be standardized and studied with objective endpoints [12,24,36,42,43,46,48].

### 6.3. Oral Agents and the Broader Pipeline

Although dermatology is not usually the main driver of antifungal research and development, it may benefit from advances generated in the broader antifungal field. Much of the current pipeline is shaped by the priorities of invasive mycoses, where mortality, hospital burden, and unmet therapeutic need strongly influence investment. However, these developments remain relevant to dermatology through cross-learning in antifungal mechanisms, resistance surveillance, diagnostic capacity, and safety considerations that are pertinent to prolonged outpatient therapy for keratinized-tissue infections [1,3,40,41,55,56].

The World Health Organization Fungal Priority Pathogens List has helped formalize antifungal resistance as a global public health and research priority. Even though its primary emphasis is not superficial mycoses, its downstream implications—greater attention to surveillance, diagnostic capacity, coordinated research, and development—are still relevant to dermatology, particularly in the context of emerging terbinafine-resistant dermatophyte infections and the expanding clinical importance of *Trichophyton indotineae* [1,3,4,5,6,8,9,40,56].

For keratinized tissues, oral innovation matters most when it improves one or more clinically relevant domains: activity in resistant settings, tolerability during prolonged courses, manageable drug–drug interaction profiles, and pharmacologic characteristics that support effective treatment of nail and skin disease when topical therapy alone is insufficient or when class-switching becomes necessary [3,4,13,14,31,32,33,39,40,41]. In this sense, the value of oral innovation in dermatology is not simply the arrival of new drugs, but the expansion of therapeutic flexibility in difficult, resistant, or recalcitrant disease [3,40,41].

Regional drug development also illustrates that clinically meaningful innovation may emerge outside globally dominant pipeline narratives. Fosravuconazole L-lysine ethanolate, developed and approved in Japan for onychomycosis, is a useful example of how pharmacokinetic optimization and region-specific development pathways can generate additional oral options for nail disease, even when broader dermatology-oriented innovation appears limited [55]. This point also emphasizes that antifungal advances are not globally uniform, and dermatologic practice may evolve through region-specific therapies, local approval pathways, and resistance epidemiology rather than through a single universal pipeline [1,3,40,41,55].

## 7. Discussion

This narrative review highlights that difficult-to-treat dermatophytosis should be interpreted through both microbiological and clinical considerations. Although terbinafine-resistant *Trichophyton* lineages, particularly *Trichophyton indotineae*, have changed the epidemiology and therapeutic expectations of dermatophytosis, genetic resistance explains only part of the clinical failure spectrum. In routine dermatology practice, persistence or relapse after apparently appropriate therapy may also reflect diagnostic uncertainty, corticosteroid-modified disease, inadequate regimen execution, reinfection, host vulnerability, and barrier-limited drug exposure in keratinized tissues. This distinction is clinically important because different drivers of failure require different corrective actions: true resistance may justify species-level identification, susceptibility testing, and rational class switching, whereas recalcitrance often requires confirmation of fungal disease, phenotypic classification, optimization of exposure and adherence, reservoir control, and measures that improve drug access and reduce protected fungal burden in high-barrier disease.

## 8. Limitations

This review has several limitations. First, it is a narrative review and did not use a systematic search strategy, formal risk-of-bias assessment, or meta-analytic methods. Therefore, the conclusions should be interpreted as an evidence-informed synthesis rather than graded guideline recommendations. Second, the available literature on resistant and recalcitrant dermatophytosis is heterogeneous, with variability in diagnostic methods, species identification, antifungal susceptibility testing, treatment regimens, and outcome definitions. Third, evidence supporting biofilm-like or aggregate behavior in dermatophyte infections is stronger for selected experimental, nail, and compact fungal mass contexts than for all dermatophyte infections in vivo. Fourth, the proposed stewardship framework has not yet been prospectively validated in clinical practice. Future studies should evaluate whether structured diagnostic and therapeutic stewardship reduces relapse, unnecessary antifungal exposure, treatment failure, and emergence of resistance.

## 9. Conclusions

Dermatophytosis is increasingly encountered as a recalcitrant clinical syndrome in which treatment failure cannot be attributed to antifungal resistance alone. True microbiological resistance, particularly terbinafine resistance associated with *Trichophyton indotineae* and other resistant *Trichophyton* lineages, must be distinguished from pseudo-resistance driven by diagnostic delay, corticosteroid-modified disease, inadequate regimen execution, reinfection, and barrier-limited drug exposure in keratinized tissues. A dermatology-centered stewardship approach should therefore move beyond reflexive drug switching and prioritize confirmation of fungal disease, careful phenotypic and anatomic classification, recognition of resistance-risk contexts, optimization of dose, duration, vehicle, and adherence, and control of persistent reservoirs. In nail disease and other high-barrier presentations, source-control strategies and topical delivery optimization are essential complements to systemic or topical antifungal therapy. Framing difficult dermatophytosis through the dual lens of resistance and recalcitrance may improve clinical decision-making, reduce unnecessary antifungal pressure, and support more rational, barrier-aware management of skin and nail dermatophyte infections.

## Figures and Tables

**Table 1:** Major Drivers of Recalcitrance in Dermatophytosis.

Driver of Recalcitrance	Clinical Clues	Evidence Base and Rationale	Clinically Oriented Action
TRUE ANTIFUNGAL RESISTANCE/RESISTANT DERMATOPHYTE LINEAGE	Persistence despite adequate therapy; extensive/inflammatory tinea; persistent mycologic positivity; *T. indotineae* risk.	Resistant dermatophytes, especially *T. indotineae*, are recognized causes of refractory disease [3,9,13,39,40].	Confirm active infection and adequate exposure before labeling resistance. Consider culture/speciation ± susceptibility testing in persistent or epidemiologically high-risk cases.
MISDIAGNOSIS/TINEA INCOGNITO	Atypical morphology, blunted border activity, eczema- or psoriasis-like lesions, extensive spread, prior corticosteroid	Reviews on tinea incognito and corticosteroid misuse consistently show delayed recognition, altered morphology, broader anatomic spread, and inappropriate retreatment cycles [10,11,25,26].	Stop inappropriate corticosteroid exposure, confirm fungal disease with KOH/mycology, and reclassify the phenotype before escalating antifungal therapy.
INADEQUATE DRUG EXPOSURE IN KERATINIZED TISSUES	Nail disease, hyperkeratosis, onycholysis, thick keratin, or slow partial response.	Keratinized barriers can limit drug access to the fungal niche [12,29,30,33,42,46,47].	Optimize site-specific exposure. Address hyperkeratosis/onycholysis and consider longer therapy or topical–systemic combinations before switching class.
COMPACT FUNGAL AGGREGATE	Dense subungual streak/patch or compact mass; limited response despite appropriate therapy.	Compact fungal masses may limit penetration and sustain recalcitrance [31,32,33,42,43,48,49].	Improve drug access with debridement, fenestration/drilling, keratolysis, or selected adjuncts plus follow-up.
REGIMEN INCONSISTENCY	Interrupted courses; multiple prior treatments; transient response then relapse; unclear history; OTC or steroid-combination use.	Non-standard or incomplete exposure contributes to persistence [17,18,24,25]	Review prior/current products, dosing, duration, adherence, self-medication, and steroid-combination use. Simplify therapy before repeated empiric switching.
EXTENSIVE CUTANEOUS DISEASE	Widespread *tinea corporis/cruris*, inflammatory or recurrent disease, multiple sites, follicular or hair-bearing involvement	Treatment intensity should match phenotype and recalcitrance risk [14,39,40,46].	Use topical-first therapy for limited uncomplicated disease. Consider systemic therapy or escalation for extensive, inflammatory, follicular, scalp-associated, or recalcitrant disease.
RELAPSE DRIVERS	Recurrence after partial response; household clustering; tinea pedis/nail disease; fomites or repeated exposure.	Relapse may reflect reservoirs or reinoculation rather than drug failure [14,17,18,22,24,27,42].	Screen for reservoirs and shared exposures. Treat concomitant disease and address prevention before labeling recurrence as resistance.
DIAGNOSTIC UNDER-ESCALATION	Multiple failed courses; no mycologic confirmation; no species-level clarification; repeated empiricism.	Selected recalcitrant cases may require culture, molecular identification, or susceptibility testing [1,3,37,39,40,46,48].	Confirm disease, define phenotype, and escalate diagnostics only when results may change management.
HOST VULNERABILITY	Diabetes; barrier disruption; neuropathy/vascular disease; recurrent or clinically significant foot disease.	Nail and foot mycoses may contribute to complications in high-risk patients [20,21,22,42].	Confirm and treat actively. Assess feet, treat concomitant disease, and avoid therapeutic inertia.

Clinical actions are based on an evidence-informed narrative synthesis of indexed epidemiologic, mechanistic, diagnostic, and treatment literature and are intended to support, not replace, formal graded guideline recommendations.

## Data Availability

No new data were created or analyzed in this study. Data sharing is not applicable to this article.

## Abbreviations

CYP51	cytochrome P450 lanosterol 14-alpha-demethylase
CYP51B	cytochrome P450 lanosterol 14-alpha-demethylase B
EUCAST	European Committee on Antimicrobial Susceptibility Testing
GBD	Global Burden of Disease
KOH	potassium hydroxide
MIC	minimum inhibitory concentration
MOB-015	topical terbinafine 10% solution
PCR	polymerase chain reaction
SQLE	squalene epoxidase
WHO	World Health Organization
